# Ethical issues in scientific publication

**DOI:** 10.4103/0019-5413.65133

**Published:** 2010

**Authors:** Anil K Jain

**Affiliations:** *Editor, Indian Journal of Orthopedics and Department of Orthopedics, University College of Medical Sciences and GTB Hospital, University of Delhi, Dilshad Garden, Delhi - 110 095, India E-mail: editor@ijoonline.com, dranilkjain@gmail.com*

Medical science grows with the pooling of scientific evidences to solve a clinical problem. A well-organized clinical or experimental research is a painstaking process. The submission of an article for publication is the final stage of long planning, execution of research, tedious analysis, and final preparation of the document.^1^ One must ensure at all stages that the data is submitted honestly. Ethics taken from the Greek word ‘ethikos,’ is defined as a set of principles or a system of right conduct. Ethical conduct is important for any sphere of life. Ethical issues are much more important in medical research and publication as they directly affect the suffering humanity. They are important during the execution and reporting of a research, reviewing an article, and for journal editors.

Research misconduct is defined by the Royal College of Physicians of Edinburgh, “as any behavior by a researcher, whether intentional or not, that fails to scrupulously respect high scientific and ethical standards. Various types of research misconduct include fabrication or falsification of data, plagiarism, problematic data presentation or analysis, failure to obtain ethical approval by the Research Ethics Committee or to obtain the subject’s informed consent, inappropriate claims of authorship, duplicate publication, and undisclosed conflict of interest”.^2^ When an article is submitted, it undergoes a chain of events from authors to reviewers, and then to editors for decision and for copy editing and scientific editing. The objective of this presentation is to discuss, how breach of ethical conduct can be prevented, who can perform a misconduct, and how it can be prevented.

During the past decade, there has been a gradual erosion of ethical principles that guide scientific research as well as writing and publication. These changes have taken place during an era where professionalism has also declined and physicians are losing control of their practices to government and corporate sectors. Thus, a growing commercialization of research with its effects on the ethical conduct of researchers and the advancement of scientific knowledge are of concern today and need serious thought. The misconduct in research and publication not only affects other authors, but reviewers and editors also. However the worst sufferer is the patient. Misconducts, whether done intentionally or through ignorance, have the same consequence. There is no difference in the seriousness of misconduct if it is done through ignorance.

The World Association of Medical Editors (WAME) has given guidelines on the publication ethics policies for medical journals on various issues such as, study design and ethics, authorship, peer review, editorial decisions, plagiarism, and how to respond to an allegation of possible misconduct.^2^ The committee on publication ethics issues a flow chart to deal with various misconducts. What is to be done if one suspects redundant publication or plagiarism or a fabricated data in a submitted or published article, or an author’s request for change of authorship, or if an editor suspects a ghost or guest or a gift authorship, or an undisclosed conflict of interest, or a reviewer appropriating the author’s idea, or the editor has shown a misconduct.^3^ Ethical misconduct could be a redundant publication, author dispute, duplicate publication, data fabrication, plagiarism or animal welfare or human welfare concerns, or conflict of interest or submission irregularities.

The authorship conflict arises when a person complains that he is a contributor to the research and his name has not been included in authors for the manuscript, or already an author informs that his consent is not being obtained or a corresponding author requests for addition of an extra author or removal of an existing author before or after publication. According to the International Committee of Medical Journal Editors (ICMJE) guidelines, anyone who has made substantial contribution to the conception, design or acquisition of data or analysis and interpretation of data, drafting or revising the article for intellectual contents, or participated in the final approval of the version to be published is entitled to be an author.^4^ Gift authorship is when an author is included just because of seniority or because he/she is a colleague or wife/husband or son/daughter, or son-in-law/daughter-in-law of the author, to increase the number of publications. The supply of patients, reagents, biological specimens or illustrations, helping in data collection, supplying funds or space or being the head of a department of an institute does not make a person eligible to become an author. This problem can be prevented if the authorship is decided in the beginning of the study. The journals safeguard themselves by asking the authors to submit a checklist including the criteria for authorship.

## DUPLICATE SUBMISSION

The submission or publication of an article by two journals that are identical or overlap substantially with or without acknowledgment to another are called duplicate publications. The authors are asked to give an undertaking that the manuscript is not submitted elsewhere and not under consideration for publication elsewhere. Publication of articles that have similar hypothesis, sample characteristics, methodology, results, and conclusion of a published article is unethical.^1^ Such articles may have the same authors or may be different authors without the knowledge of the initial authors.^5^ It is done to increase the number of publications or are more common with the pharmaceutical industry. 6 When an article is republished as a part or parts of an already published article, it is labeled as a redundant publication. The publication of a single data set into multiple articles is called salami slicing.^6^ Such publications are unethical as it wastes the time of reviewers, occupies the valuable space of published scientific data, and such unnecessary over-emphasized publication inflates scientific literature with flawed meta-analysis for no benefit other than to the author. They are serious violations of the copyright law. If one such article is found before publication it is rejected or if found after publication, then a notice is printed in the next issue about the duplicate publication and the said article is withdrawn. Sometimes such publications are acceptable when the manuscript is a guideline or in another language, and the article is intended for different readers. Such publications are prepared with concurrence from editors of both journals and respecting the priority of the primary publication. If a manuscript is based on the same study and their analytic approach is different, then it can be published as two articles or a single article with a commentary. Duplicate publication of the same data should be avoided as publication bias distorts the literature and the meta-analysis may be skewed in the duplicate data.

## FALSIFICATION/FABRICATION

Fabrication of data is a recording of fictitious data when none exists and falsification is the manipulation of data or experimental procedures to produce a desired outcome or to avoid a complicating or inexplicable result. Selective reporting of data is very common in the pharmaceutical industry-sponsored research, where few significant effects of a particular drug are selectively reported ignoring the large number of nonsignificant effects. Thus drugs appear more potent than they really are. This misconduct is not very uncommon. A meta-analysis of surveys exists where scientists were asked directly if they have committed or know of a colleague who has committed research misconduct. A pooled average of 1.97% admitted to have fabricated, falsified or modified data or results at least once and up to 33.7% admitted questionable research practices, for other colleagues this figure rose to 14.12% for falsification and up to 72% for questionable research practices.^7^ Thus over-publication or under-publication can give rise to misleading conclusions from the meta-analysis. This type of misconduct dilutes the genuine research done by other authors and researchers. It not only wastes the time of other researchers who have planned further studies based on studies with fabricated data, but also consumes the limited research resources. The conclusions drawn on the basis of such studies may affect further clinical practice, and when such studies are exposed, the trust of the public on medical research is shaken.

## PLAGIARISM

The plagiarism is defined as copying ideas, passages of text from someone else, and using them as if they were ones own. According to a committee of publication ethics guidelines plagiarism ranges from unreferenced use of others’ published and unpublished ideas to the submission of a complete article under a new authorship.^3^ In a questionnaire-based study, to assess the knowledge and perceptions of plagiarism among medical students and facility members, a general lack of information regarding plagiarism was observed.^8^ The Indian Journal of Orthopedics rejects almost 10 - 15 plagiarized manuscripts every year, where paragraphs after paragraphs are found to be taken verbatim from an already published article, without referencing the original manuscript. Plagiarism is easier to commit with the progress in the field of the internet.^9^

Whenever a manuscript is submitted, the editorial teams perform a literature search and most of the time it is possible to detect an already published text, passage. Detection of plagiarism used to be difficult in the past. The web-based data is easier to be detected.^9^ Experienced editorial board members are able to suspect even after reading of a manuscript. The principle author is responsible for this misconduct, whether it is intentional or inadvertant. Intentional violation generally has more severe consequences, and an open and honest reply, with appropriate documentation, can help to demonstrate that any error made was not intentional.

## ETHICAL RESPONSIBILITIES OF EDITORS AND REVIEWERS

The editors and reviewers also have to follow ethics. The authors have submitted their most valuable piece of research work. The reviewers and editors are expected to maintain confidentiality and not to misappropriate ideas or text. They should submit the reviews in a timely manner. Conflict of interest of any kind whether it is financial, personal or any other kind should be disclosed. The review should only be done only when the reviewer feels that he is competent to do. If a reviewer has got the review performed by any other colleague, who is an expert on the subject, then the editor should be informed about this.^10^ The editors and editorial team members should maintain confidentiality of the research submitted and should not misappropriate the data.

Specific guidelines on the ethics of healthcare-related research have been made and revised by a number of international bodies, including the World Medical Association (WMA). A central document, the Declaration of Helsinki, put together in 1964, by the World Medical Association is the earliest directing principle of human research, formed in an effort to ensure that medical research would follow ethical rules of practice and be of benefit to both researchers and research subjects.^11^ WAME, established in 1995, is a nonprofit voluntary association of editors of peer-reviewed medical journals, from countries throughout the world, who seek to foster international cooperation among the education of medical journal editors. WAME facilitates worldwide cooperation and communication among editors of peer-reviewed medical journals, to improve editorial standards, to promote professionalism in medical editing through education of self-criticism and self-regulation, and encourage research on the principles and practice of medical editing.^2^

The Committee on Publication Ethics (COPE), a voluntary body, was founded in 1997, to address breaches and code of conduct for research and publication ethics for biomedical journals. It provides a discussion forum and advice for scientific editors and aims to find practical ways of dealing with the issues, to develop good practices. It attempts to define the best practice in the ethics of scientific publishing by providing guidelines for authors, editors, editorial board members, readers, owners of journals, and publishers. They address: study design and ethical approval, data analysis, authorship, conflict of interests, the peer review process, redundant publication, plagiarism, and duties of editors, media relations, advertising, and how to deal with misconduct. When any such kind of misconduct is reported, the editors act as suggested by the COPE guidelines.^3^ The jest of these quidelines is that a serious attempt is made to define misconduct. The author is given an oppurtunity to explain whether plagiarism or other misconduct is an honest error. However, if the response from author is unsatisfactory and intentional misconduct cannot be ruled out, the manuscript is rejected if it is under the process of review. However, if the article is already published, then it is retracted with a notice published in the next issue. The retraction of an already published article becomes a printed notice in the journal and that is very awkward for the author. The author’s institution is informed of the misconduct and journals reply to the academic institution to take action. If repeated misconduct is encountered from the same person, then the other journals are also informed.

The quest of alleviating the pain of the suffering humanity is the driving force to conduct research; hence, the research should be planned, executed, analyzed and published in an honest form. Ideally one should not require a law or guidelines to prevent misconduct in research and publication, with self-restraint being the best method to conduct an honest and credible research and publication.
